# Nano silica’s role in regulating heavy metal uptake in *Calendula officinalis*

**DOI:** 10.1186/s12870-024-05311-1

**Published:** 2024-06-25

**Authors:** Maryam Samani, Yogesh K. Ahlawat, Ahmad Golchin, Hossein Ali Alikhani, Ahmad Baybordi, Sadhna Mishra, Özhan Şimşek

**Affiliations:** 1 Soil Science Department, Faculty of Agriculture, University of Zanjan, Zanjan, Iran; 2 Sharda School of Smart Agriculture, Sharda University, Agra, Uttar Pradesh 282007 India; 3 Centre of Research Impact and Outreach, Institute of Engineering and Technology, Chitkara University, Rajpura, Punjab 140401 India; 4 Centre of Research Impact and Outreach, Chitkara University, Baddi, Himachal Pradesh 174103 India; 5 Soil Science Department, Faculty of Agriculture, University of Tehran, Tehran, Iran; 6 Soil and water Research Department, East Azerbaijan Agriculture and Natural Resources Research and Education Center, AREEO, Tabriz, Iran; 7 Faculty of agricultural sciences, GLA university, Mathura, Uttar Pradesh 281406 India; 8 Horticulture Department, Agriculture Faculty, Erciyes University, Kayseri, 38030 Türkiye

**Keywords:** Silica, Nutrients uptake, Dry matter yield, *Calendula officinalis*, Enzyme activity

## Abstract

**Background:**

Soil contamination with heavy metals poses a significant threat to plant health and human well-being. This study explores the potential of nano silica as a solution for mitigating heavy metal uptake in *Calendula officinalis*.

**Results:**

Greenhouse experiments demonstrated, 1000 mg•kg^− 1^ nano silica caused a 6% increase in soil pH compared to the control treatment. Also in 1000 mg. kg^− 1^ nano silica, the concentrations of available Pb (lead), Zn (zinc), Cu (copper), Ni (nickel), and Cr (chromium) in soil decreased by 12%, 11%, 11.6%, 10%, and 9.5%, respectively, compared to the control. Nano silica application significantly reduces heavy metal accumulation in *C. officinalis* exposed to contaminated soil except Zn. In 1000 mg.kg^− 1^ nano silica shoots Zn 13.28% increased and roots Zn increased 13% compared to the control treatment. Applying nano silica leads to increase the amount of phosphorus (P) 25%, potassium (K) 26% uptake by plant, In 1000 mg.kg ^− 1^ treatment the highest amount of urease enzyme activity was 2.5%, dehydrogenase enzyme activity, 23.6% and the highest level of alkaline phosphatase enzyme activity was 13.5% higher than the control treatment.

**Conclusion:**

Nano silica, particularly at a concentration of 1000 mg.kg ^− 1^, enhanced roots and shoots length, dry weight, and soil enzyme activity Moreover, it increased P and K concentrations in plant tissues while decreasing heavy metals uptake by plant.

## Background

In recent years, the escalating concern over environmental pollution and its detrimental effects on plant health has led to innovative approaches aimed at optimizing soil conditions. Heavy metal pollution is a significant environmental concern. Due to the persistent and highly toxic nature of these pollutants, soil contaminated with such metals poses environmental problems that affect plants, animals, and ultimately human health [1, 2]. Research has shown that plants growing in polluted environments exhibit altered metabolism [3], growth reduction [4], lower biomass production [5], and oxidative damage along with the accumulation of pollutants [6]. Consequently, there is an increasing need to manage toxic elements to protect agricultural land. Soil enzymes exhibit greater sensitivity to heavy metal stress compared to plants and animals [7], making them valuable indicators of soil conditions and quality [8]. Heavy metals also diminish the number, diversity, and activity of soil microbes, subsequently reducing the production of extracellular enzymes [9]. Elevated cadmium (Cd) concentrations significantly decrease the activity of enzymes such as catalase, urease, dehydrogenase, cellulase, and phosphatase [10]. The traditional stabilizer for immobilization of heavy metals includes lime, hydroxyapatite, zeolite, phosphates [11], bentonite [12], fly ash and red mud and so on [13]. Furthermore, new materials, such as nano-materials [14], biochar [15, 16], polymer [17] and modified material [18] are also used as a stabilizer to remediate heavy metal contaminated soils. These stabilizers can reduce the activity of heavy metals in soils at certain extent, but their specificity and long-term stability are not enough, and their influence on soil properties has not been detected, which limited their large-scale application. Therefore, it is necessary to develop a new stabilizer with strong specificity, long-term stability and few adverse effects on soil environment. Silica, which is well compatible with soils, has been used in many fields, such as the additives of lubricating oils and cement [19]. Functionalized nano-silica can improve the properties of polymer materials [20] and enhance oil recovery [21]. Silica or functionalized silica has also been used as adsorbents to adsorb heavy metals from aqueous systems and to remove them effectively [22, 23]. Some Si-based materials have also been used to remediate heavy metals in soil and alleviate the stress of heavy metals to plants [24, 25]. the application of modified nano-silica transformed Cu, Pb, and Zn to a more stable fraction in soil [26]. Nano-silica decreased the DTPA-extractable Cd in soil effectively [27]. Therefore, a new amendment, with higher stabilization efficient and lower application rate, was needed to remediate heavy metal contaminated soil.

Silica, an inorganic solid characterized by a three-dimensional network structure and a porous configuration with an extensive surface area, primarily comprises siloxane groups (Si-O-Si) internally and silanol groups (Si-OH) on the particle surface. The chemistry of these surface groups, including single-classified silanol, geminal (binary), and adjacent silanol, influences properties like adsorption, adhesion, catalytic activity, and chemical reactivity of silica [28]. Silanol groups, hydrophilic in nature, are found both on the surface and within the silica skeleton, while siloxanes exhibit hydrophobic characteristics [29].

Research suggests that silicon dioxide nanoparticles serve as effective adsorbents for removing natural pollutants and metal ions [30]. Plants receiving sufficient silicon display heightened resistance to environmental stresses and heavy metals [31]. Silica plays a crucial role in reducing the toxicity of heavy metals by impeding their absorption and transference within the plant. The use of silica has been reported to enhance the tolerance of various plant species to heavy metals by minimizing their absorption and transport. The primary mechanisms by which silicon corrects heavy metal stress in plants encompass: (1) entangling or combining metals with silicon, (2) preventing metal transfer from roots to aerial organs, (3) internal fixation of metal ions within the plant, and (4) stimulating the antioxidant system and inducing changes in cellular structure (32). Additionally, silica contributes to metal detoxification by influencing plant cell mechanisms and biochemical interactions with the external growth environment [32].

Moreover, this study explores the consequences of nano silica application on nutrient dynamics in the soil. Nutrients are vital for plant growth and health, making it crucial to understand how soil amendments like nanosilica interact with soil nutrients. Marigold (*C. officinalis*), is choosen for this study due to its widespread use in pharmaceutical, cosmetic, and culinary industries, as well as its role as a bioindicator for soil health. The response of *C. officinalis* to the nano-silica treated soil provides valuable insights into the broader implications of using such soil amendments in agricultural and horticultural practices. The effect of silica on the activity of soil enzymes has been less investigated, therefore, one of the goals of this research was investigate **(i)** The effect of nano silica on nutrients uptake by plant and enzymes activity in soils. **(ii)** stabilization efficiency of heavy metals in soils by nano silica; **(iii)** influences of nano silica on heavy metal uptake by plants.

## Methods

To investigate the efficiency of nano silica in reducing the mobility and plant availability of heavy metals lead (Pb), zinc (Zn), copper (Cu), chromium (Cr), and nickel (Ni) in the soil and also its effect on the growth characteristic and concentration of heavy metals, phosphorus (P), and potassium (K) in the marigold plant, an experiment in the completely random design (CRD) was implemented in pots and greenhouse conditions. A composite soil sample was collected from a depth of 0–15 cm in an urban park located in Tehran, Iran. The sample was air-dried, passed through a 2-mm sieve, analyzed for physico-chemical properties, and used for this study.

### Soil analysis for heavy metal

A composite soil sample was collected from a depth of 0–15 cm in an urban park located in Tehran, Iran. The sample air- dried, passed through a 2-mm sieve, analyzed for physico- chemical properties and used for this study. Soil texture was determined by the hydrometer method [33]. The pH of saturated paste (1:5) soil to water ratio (pHs) and the electrical conductivity of saturated extract (ECe) were measured using a pH meter and an EC meter respectively [34, 35]. The concentration of organic carbon in the soil (OC%) was determined by the wet combustion method using potassium dichromate and sulfuric acid [36]. Cation exchange capacity (CEC) was calculated using the sodium acetate method [37] and SSA was measured by BET. The available fractions of heavy metals in the soil were extracted by DTPA [38], 10 g of soil was added in 20 mL mixture of 0.005 mol·L^− 1^ DTPA and 0.01 mol·L^− 1^ CaCl_2_ and 0.01 mol·L^− 1^ triethylamine (pH 7.3), shaken for 2 h and then filtered through using a Whatman (No. 42) filter. For the total concentration of heavy metals in soil, the soil samples were digested using (the aqua regia digestion method), 3 g of soil was placed in a 100 ml round bottom flask with 21 ml of concentrated HCl (35%) and 7 ml of concentrated HNO3 (65%). The solution was kept at room temperature overnight before a water condenser was attached and the solution was heated to boiling point for 2 h. Added 25 ml water to the condenser before filtration of the mixture through using a Whatman (No. 42) filter. The filtered residue was rinsed twice with 5 ml of water and the solution was made up to 100 ml [39]. The heavy metal concentrations were measured by ICP-MS. All samples and analysis had 3 replicants for accuracy.

### Greenhouse study

A greenhouse pot experiment was conducted to examine the impact of nano silica on the immobilization of Pb, Zn, Cu, Ni, and Cr in soil. The experiment followed a completely randomized design with three replications. Nano silica was incorporated into samples of urban soil at varying rates of 0, 100, 200, 500, and 1000 mg.kg^− 1^. Both treated and untreated soil samples were then incubated for two months at field capacity (FC) moisture levels. Following the incubation period, marigold plants were planted in each pot. Throughout the approximately 75 day growth period, pots were irrigated with distilled water to maintain field capacity moisture levels. Daily monitoring of pot weight enabled adjustments to compensate for water loss until FC moisture was attained. Upon completion of the growth period, plant shoots and roots were harvested and transported to the laboratory, where they were washed sequentially with distilled water. Subsequently, plant height, wet weight of shoots and roots, were measured before individually placing plant samples in paper pockets for 72 h oven drying at 60℃. After drying, samples were ground, sieved, and digested with concentrated nitric acid and 30% hydrogen peroxide at 120 °C [40]. The final extracts were analyzed for heavy metal concentration using ICP-MS, while P and K concentrations were determined using a spectrophotometer (CE 292 Digital UV-Visible spectrophotometer) and a flame photometer (Jenway PFP7), respectively.

### Soil enzymes

The activities of urease, dehydrogenase and alkaline phosphatase enzymes were measured in treated and untreated soil samples according to Tabatabai and Bremner [41].

Alkaline phosphatase measurement: One gram of soil was weighed and 0.25 ml of toluene, 4 ml of phosphate buffer (pH = 11) and one ml of paranitrophenol substrate solution were added to it. The samples were incubated for one hour at 37 degrees. Then the solution was filtered with Whatman 40 filter paper and 4 ml of 0.5 M sodium hydroxide solution and 1 ml of 0.5 M calcium chloride solution were added and shaken to complete the enzyme activity. The samples were read by a spectrophotometer at a wavelength of 410 nm and calculated on (µgPNP. g^− 1^ soil h^− 1^).

Measurement of urease enzyme: 5 g of soil was treated with 0.2 ml of urea solution and after adding 9 ml of Tris buffer (pH = 9) it was incubated for two hours at 37 degrees. Then 35 ml KCl-AgSO_4_ solution (2.5 M compared to KCl and 100 mg/liter compared to AgSO_4_) was added to it. The amount of ammonium released in the existing suspension was determined by colorimetric method and after reducing the amount of ammonium in the control treatment, reported as (mg N-NH^+ 4^ gr^− 1^ soil h ^− 1^ ).

Dehydrogenase enzyme activity: 5 g of soil were treated with 5 ml of 0.6% triphenyltetrazolium chloride (TTC) solution as substrate and placed in an incubator at 25 ℃ for 16 h. After incubation for the extraction of triphenyl formazan (TPF) 25 ml of ethanol was added to the soil sample and stirred for 2 h in the dark with a shaker. Then the desired mixture was passed through Whatman 42 filter paper and the amount of light absorption of the samples at a wavelength of 546 nm was measured by a spectrophotometer and the amount of enzyme activity was reported as (µg TPF g^− 1^ soil 16 h^− 1^) [42].

### Soil amendment

Nano silica with a chemical formula SiO_2_ 99.5% purity was prepared from Pasargad Novin Chemical Company.

#### Nano sorbent evaluation instruments

To determine nano silica elemental composition, used X-ray Fluorescence (XRF), an analytical technique that uses the interaction of X-rays with a material to determine its elemental composition.

To determine crystallographic structure of nano silica used X-Ray diffraction analysis (XRD). It is a nondestructive technique that provides detailed information about the crystallographic structure, chemical composition, and physical properties of a material.

To determine the surface morphology of particles of nano silica used Scanning electron microscopy (SEM) or SEM analysis is a powerful analytical technique to perform analysis on a wide range of materials, at high magnifications, and to produce high resolution images.

To identify functional groups of nano silica used Fourier-transform infrared spectroscopy (FTIR). The FTIR analysis method uses infrared light to scan test samples and observe chemical properties.

To calculate the specific surface area of nano silica, used Brunauer-Emmett-Teller (BET) based on gas adsorption measurements. The method is suitable for analyzing a wide range of solid matrices from catalyst powders to monolithic materials.

### Data analysis

The data were analyzed using the SPSS 21.0 statistical software package and Excel 2016 for Windows. The means of three replicates were subjected to one-way ANOVA using the Duncan test at the 0.05 confidence level. The experiment was conducted based on a completely randomized design (CRD). The treatments included five levels of nano silica at levels (0, 100, 200, 500, 1000 m.kg^− 1^) in three replicates. The completely random design was chosen because the base of the samples was equal and the only variable factor was the treatment level of nano silica. The number of treatments and the number of repetitions were balanced CRB. Mean comparison was done through Duncan’s test at 5% confidence level.

## Results

### Soil characteristics

Some physicochemical properties of the soil used in this study are summarized in Table 1. According to our findings, the studied soil had a Silty Loam texture, relatively neutral pH, and low EC and OC content.

**Table 1:** Physical and chemical properties of the soil used in this study

Texture	Silty loam
Sand (%)	38
Silt (%)	51
Clay (%)	11
pH	7.47
EC (dS m^− 1^)	0.45
CEC (Cmol_c_ kg^− 1^)	14.8
OC (%)	0.6
SSA(m^2^g^− 1^)	19.63
Available Pb (mg kg ^− 1^)	7.54
Available Zn (mg kg ^− 1^)	27.12
Available Cu (mg kg ^− 1^)	7.75
Total Pb (mg kg ^− 1^)	59.82
Total Zn (mg kg ^− 1^)	200.95
Total Cu (mg kg ^− 1^)	61.5

### Characteristics of nano silica used in this study

#### Chemical analysis of nano silica by XRF

The results of chemical analysis of nano silica by XRF are shown in Table 2. Silica nanoparticle has more than 99% silica dioxide and the impurities in it include iron (F) and sodium (Na) respectively with amounts less than 20 and 50 mg.kg^− 1^, calcium (Ca) and titanium (Ti) respectively with values less than 70 and 120 mg.kg^− 1^.

**Table 2:** Chemical compounds of nano silica based on XRF analysis

SiO2	Ca	Na	F	Ti
(%)	mg.kg^− 1^			
> 99	< 70	< 50	< 20	< 120

#### XRD results of nano-silica

**Fig. 1:**
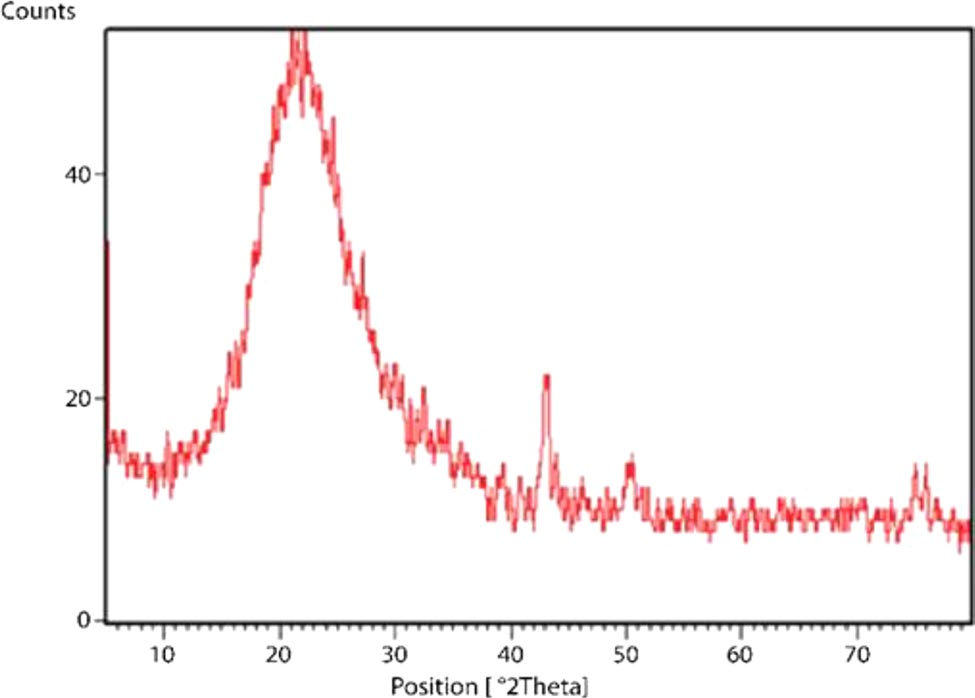
The XRD pattern of nano silica. Crystallin structure in tetragonal crystal system. Intense peaks at 22.15 and 44.3 angles indicate the presence of SiO_2_ crystal structure in the tetragonal crystal system

The XRD pattern of silica nano absorbent is shown in Fig. 1. Intense peaks at 22.15 and 44.3 angles indicate the presence of SiO_2_ crystal structure in the tetragonal crystal system. Parameters a, b and c are determined as 4.7, 4.7 and 7.4 respectively. Among other crystallographic parameters of this material, we can mention alpha, beta and gamma, all of which are 90 degrees (Fig. 1).

#### The SEM results of nano silica

**Fig. 2:**
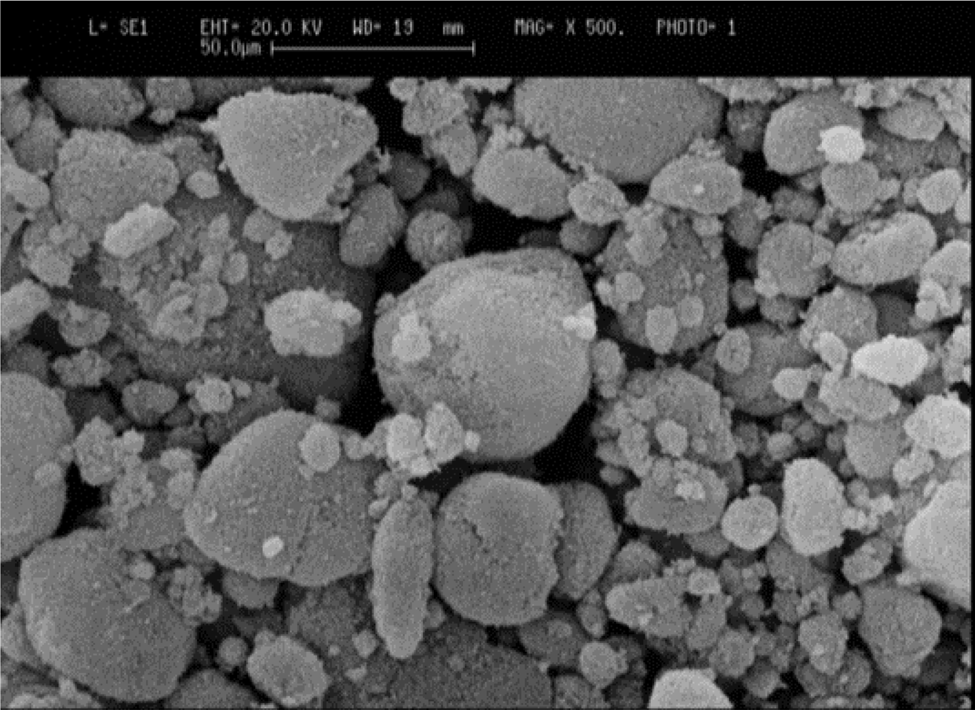
Nano silica SEM image. Silica nanoparticles have a spherical shape and the voids in the shape indicate the presence of places for the absorption of heavy metals. These empty spaces increase the potential of silica nanoparticles to absorb heavy metals

The surface morphology of silica particles is shown in Fig. 2 using an electron microscope (SEM). Silica nanoparticles have a spherical shape and the voids in the shape indicate the presence of places for the absorption of heavy metals. These empty spaces increase the potential of silica nanoparticles to absorb heavy metals.

#### The FTIR results of the nano-silica

**Fig. 3:**
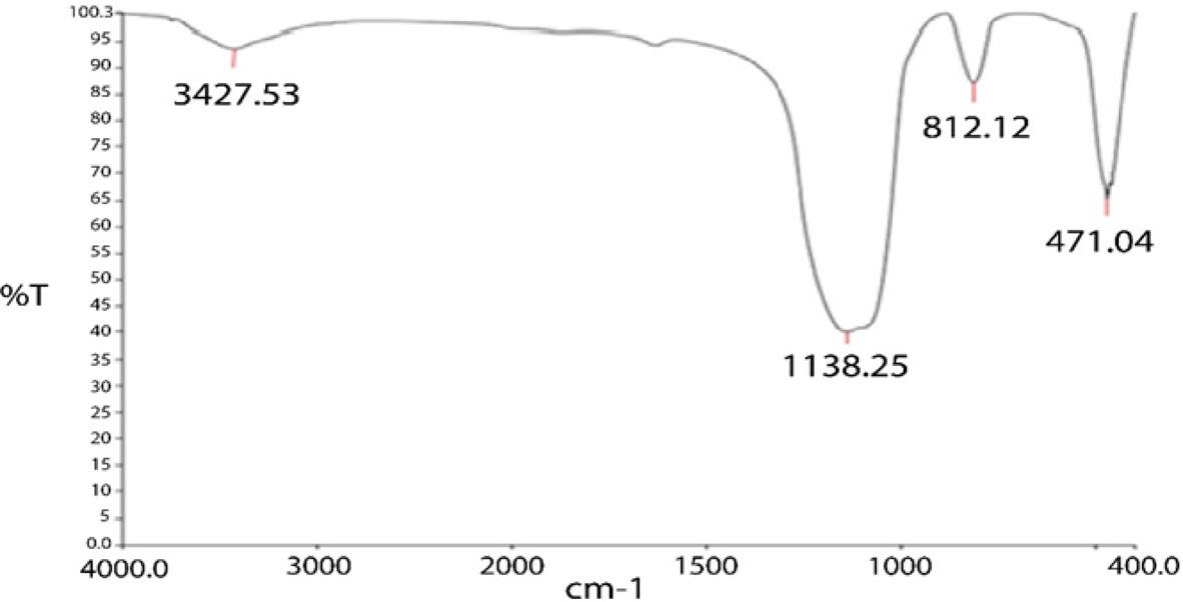
Nano silica FTIR image. The strong peaks in the region of 471.04, 812.12, and 1138.25 cm^− 1^ are related to the asymmetric stretching vibrations of siloxane groups (Si-O-Si). The peak in the region of 3427.55 cm^− 1^ corresponds to the vibrational stretching of the O-H group, which overlaps with the silanol (Si-OH) group

FTIR analysis was used to determine the functional groups affecting surface absorption. The FTIR spectra of silica nanoparticles are shown in the Fig. 3. The strong peaks in the region of 471.04, 812.12, and 1138.25 cm^− 1^ are related to the asymmetric stretching vibrations of siloxane groups (Si-O-Si). The peak in the region of 3427.55 cm^− 1^ corresponds to the vibrational stretching of the O-H group, which overlaps with the silanol (Si-OH) group [43, 44].

#### The specific surface area of the nano silica by BET technique

**Fig. 4:**
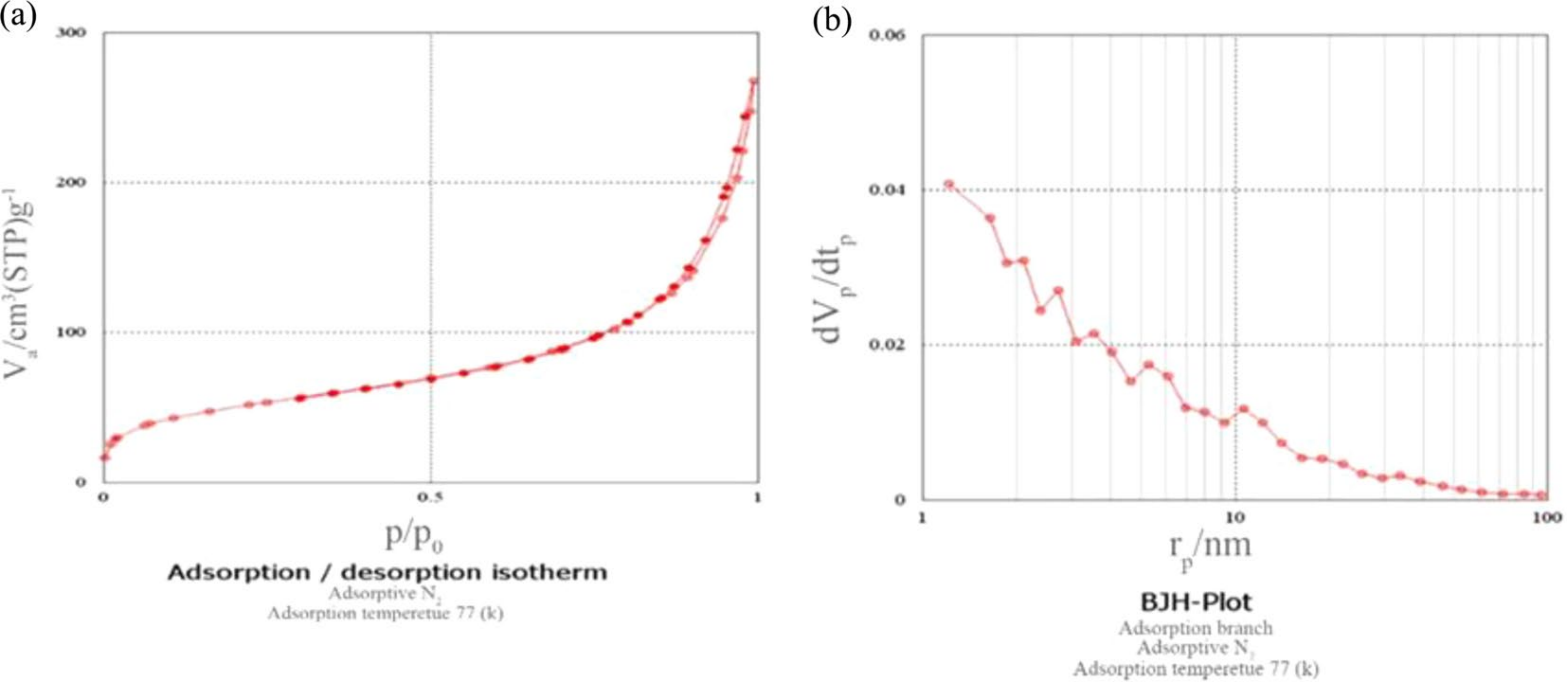
(**a**) Absorption/desorption diagram of nano silica, Specific surface area 179.68 m^2^.gr ^− 1^, mesoporous structure. (**b**) BJH diagram of nano silica, the total volume of the holes was 0.397 (cm^3^.gr^− 1^) and the diameter of the holes was 2.42 nm

Based on the results of BET analysis, the absorption and desorption curve of nano silica is type IV, which indicates the mesoporous structure of silica. Silica nanoparticles have a specific surface area of 179.68 (m^2^.gr^− 1^) and the percentage of porosity is 93.95% (Fig. 4a). The mesopore volume and mesopore diameter are obtained from the BJH curve. According to the BJH curve, the total volume of the holes was 0.397 (cm^3^.gr^− 1^) and the diameter of the holes was 2.42 nm (Fig. 4b).

### The effects of nano-silica on the concentrations of DTPA extractable metals and soil pH

The results of variance analysis of the data showed that different levels of silica nano absorbent had a significant effect at the probability level of 1% (*p* < 0.01) on the concentration of heavy metals extractable with DTPA and on soil pH (Table 3).

**Table 3:** The results of variance analysis of different levels of nano silica on DTPA extractable heavy metals and pH

MS DTPA extractable soil heavy metals							
SOV	df	**Pb**	**Zn**	**Cu**	**Ni**	**Cr**	**pH**
Treatment	4	0.49**	5.36**	0.45**	0.23**	0.47**	0.1^**^
Error	10	0.03	0.004	0.011	0.007	0.012	0.008
COV (%)	-	2.42	0.24	1.42	1.4	1.21	1.47

** and * are significant at 1% and 5% levels, respectively, and ns is not statistically significant

**Fig. 5:**
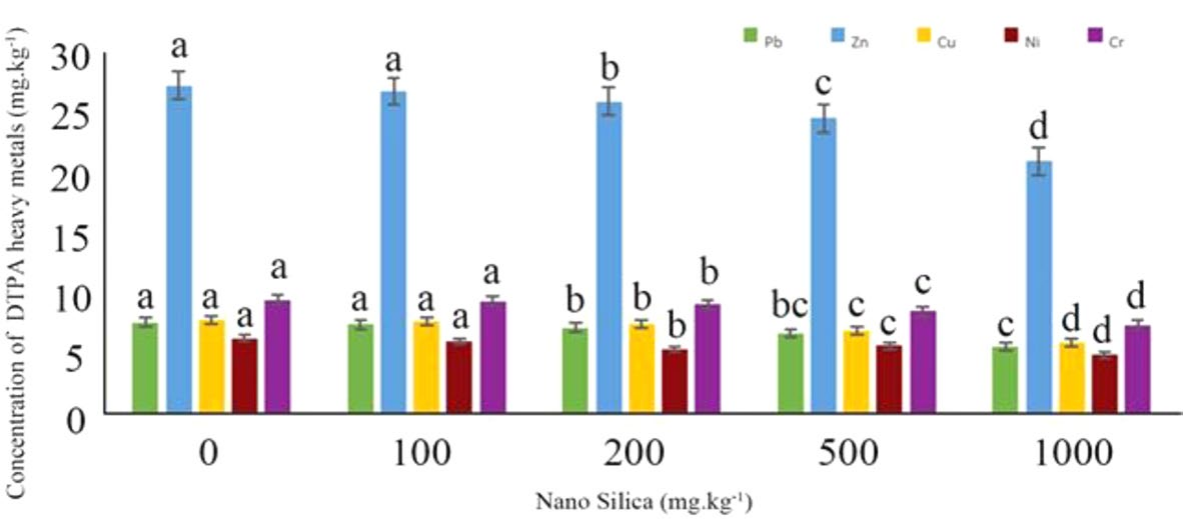
The effect of different level of nano silica on concentration of DTPA heavy metals. The highest available concentration of metals was in the control treatment and the lowest available concentration of metals was in the 1000 mg.kg^− 1^ treatment

The results of the average comparison of the effects of the amount of nano silica on the concentration of DTPA extractable heavy metal showed that the highest available concentration of metals was in the control treatment and the lowest available concentration of metals was in the 1000 mg.kg^− 1^ treatment (Fig. 5).

The concentrations of Pb DTPA extractable ranged from 6.63 to 7.54 mg.kg^− 1^, the lowest concentration observed at 6.63 mg.kg^− 1^ and the highest at 7.54 mg.kg^− 1^. When treated with 1000 mg.kg^− 1^ of silica nano absorbent, there was a 12% decrease in available Pb compared to the control. The control treatment showed the highest concentrations of DTPA-extractable Zn and Cu, at 27.12 and 7.75 mg.kg^− 1^ respectively. In contrast, Zn and Cu DTPA- extractable in 1000 mg.kg^− 1^ nano silica were in lower concentrations 24.08 and 6.85 mg.kg^− 1^ respectively, representing an 11% decrease for Zn and 11.6% for Cu compared to the control. For Ni and Cr, the highest available concentrations in the control treatment were 6.24 and 9.42 mg.kg^− 1^ respectively, whereas in the 1000 mg.kg^− 1^ treatment, were 5.61 and 8.52 mg.kg^− 1^ respectively, indicating a decrease of 10% for Ni and 9.5% for Cr compared to the control. The highest effect of nano silica in reducing the available concentration of heavy metals in the soil tested, was Pb > Cu > Zn > Ni > Cr (Table 4).

**Table 4:** Comparing the averages of the effect of different levels of silica nano absorbent on the concentration of metals extractable with DTPA and soil pH

	DTPA heavy metals (mg.kg^− 1^)					
Nano silica(mg.kg^− 1^)	Pb	Zn	Cu	Ni	Cr	pH
**0**	5.54a	27.12 a	7.75 a	6.24 a	9.42 a	7.43 c
**100**	7.54 a	27.1 a	7.73 a	6.23 a	9.41 a	7.7 b
**200**	7.16 b	25.92 b	7.4 b	6 b	9.08 b	7.79 a
**500**	6.86 bc	24.95 c	7.13 c	5.77 c	8.76 c	7.87 a
**1000**	6.63 c	24.08 d	6.85 d	5.61 d	8.52 d	7.88 a

‘Averages that have at least one letter in common are not statistically significant’

**Fig. 6:**
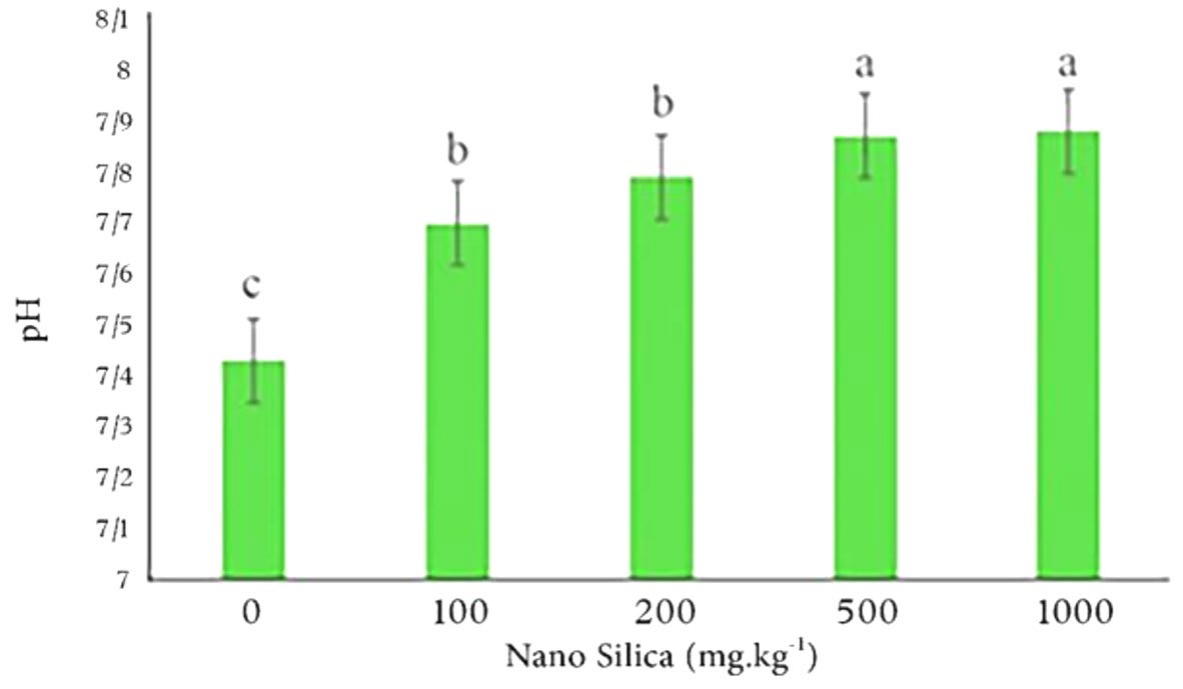
The effect of different level of nano silica on soil pH. 1000 mg.kg^− 1^ nano silica caused a 6% increase in soil pH compared to the control treatment

The results indicated that 1000 mg.kg^− 1^ nano silica leads to 6% increase in soil pH compared to the control treatment (Fig. 6).

**Fig. 7:**
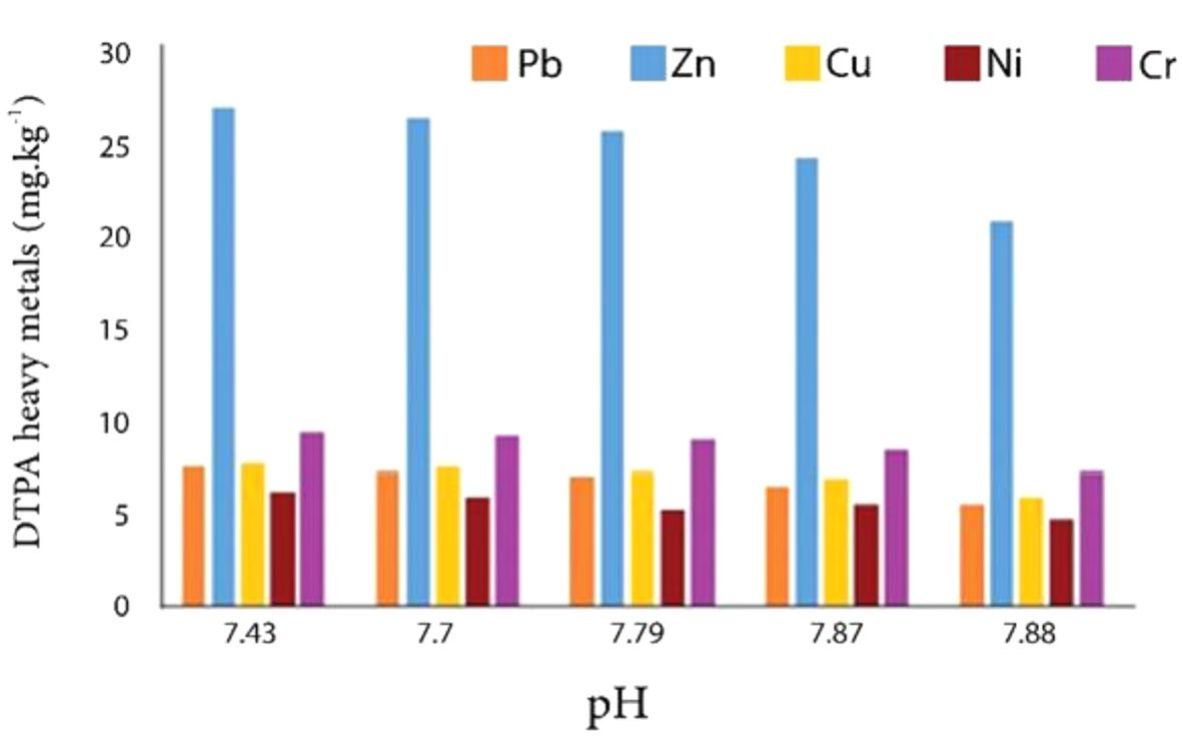
The effect of pH on DTPA heavy metals in examined soil. By increasing pH the concentration of DTPA heavy metals decreased

The results showed by increasing the soil pH, the DTPA extractable concentrations of heavy metals decreased (Fig. 7).

### The effect of different amounts of nano silica on the shoots and roots height, dry and fresh weight of roots and shoots of *C. Officinalis*

The results of the variance analysis of the effect of different nano silica treatments on the fresh and dry weight of roots and shoots, the average height of shoots and roots of *C. officinalis* showed that the effect of different levels of nano silica on fresh and dry weight of shoots and roots was not significant. The effect of different levels of nano silica on shoots length, roots length and roots dry weight was significant at the probability level of 1% (*p* < 0.01) (Table 5).

**Table 5:** The results of analysis of variance of the effect of different levels of nano silica on the height of shoots and roots, dry and wet weight of roots and shoots of *C. officinalis*

MS							
SOV	df	Shoots wet weight	Shoots dry weight	Roots wet weight	Roots dry weight	Shoots height	Roots height
		g.pot^− 1^				Cm	
Treatment	4	1.59ns	0.251ns	0.34ns	0.039**	0.143**	0.012**
Error	10	3.26	0.64	0.449	0.006	0.001	0.001
COV (%)	-	2.97	12.96	4.47	5.65	0.34	0.28

** and * are significant at 1% and 5% levels, respectively, and ns is not statistically significant

**Fig. 8:**
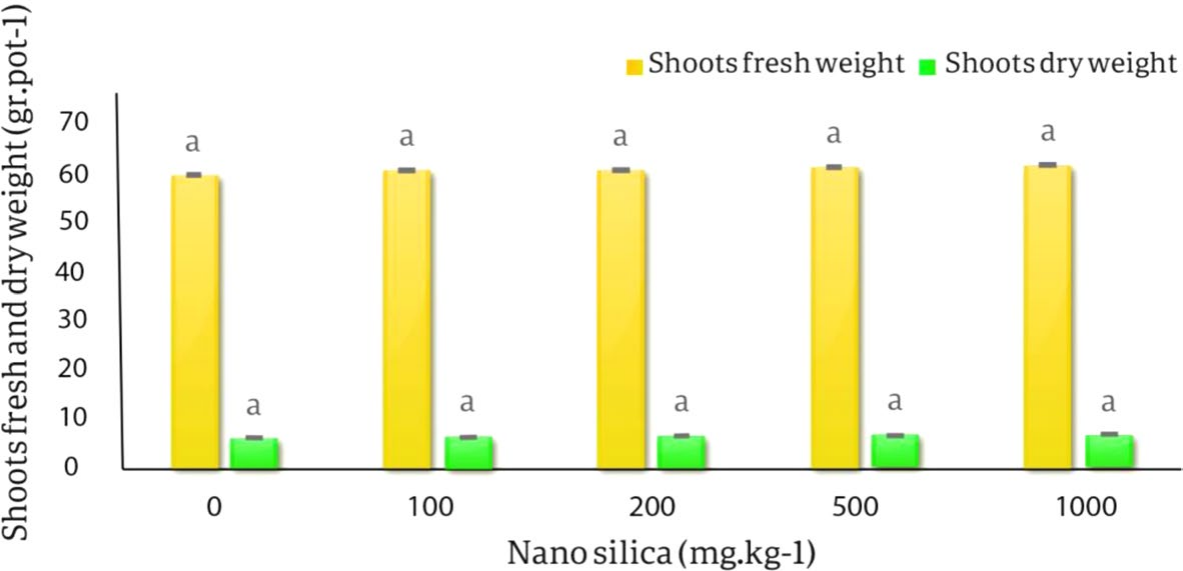
The effect of nano silica on fresh and dry shoots weight. The lowest fresh and dry weight were related to control treatment and the highrst fresh and dry weight were related to 1000 mg.kg-1 nano silica by 3.27% and 12.5% compared to control treatment, respectively

The results of averages comparing of the effect of different levels of nano silica on the fresh weight of shoots showed that the lowest amount of fresh weight of shoots was in the control treatment 59.5 gr.pot^− 1^ and the highest amount of fresh weight of shoots was in the 1000 mg.kg^− 1^ of nano-silica and equal to 61.45 gr.pot^− 1,^ which increased by 3.27% compared to the control treatment, but this increase was not statistically significant. The lowest dry weight of shoots was related to the control treatment and was 5.81 gr.pot^− 1^, and the highest amount of dry weight of shoots was 6.54 gr.pot^− 1^ in the treatment of 1000 mg of nano silica. kg^− 1^ soil. There was an increase of 12.5%, compared to the control but this increase was not statistically significant (Fig. 8).

**Fig. 9:**
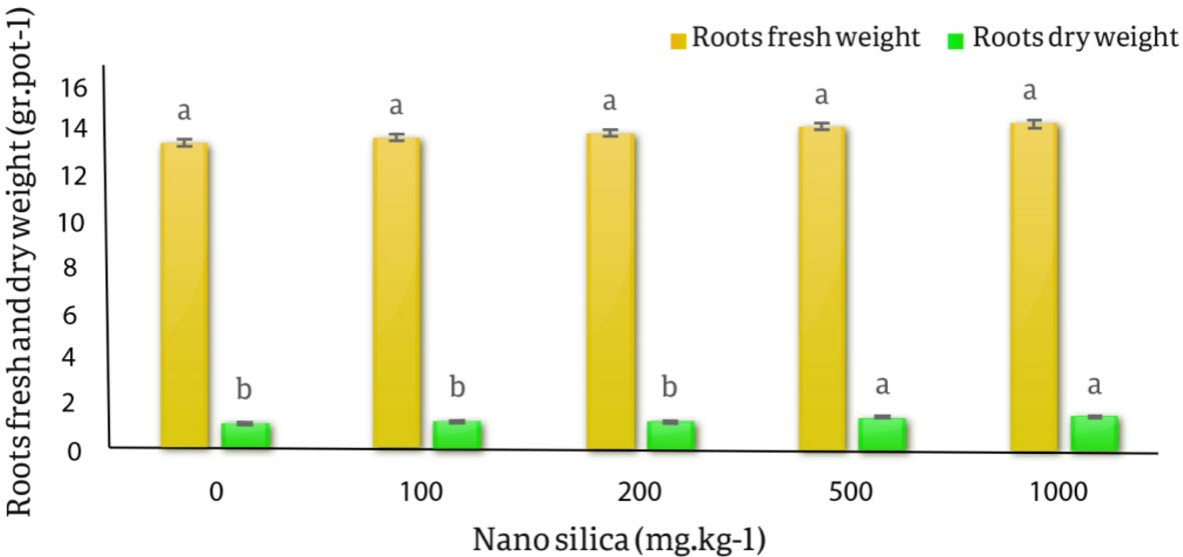
The effect of nano silica on fresh and dry roots weight. In 1000 mg.kg^− 1^ nano silica fresh roots weight increased 5.97% and dry roots weight increased 20.8% compare to the control treatment

The results of the averages comparison of the effect of different levels of nano silica on the wet and dry weight of the roots of the *C. officinalis* showed that the lowest wet weight of the roots was related to the control treatment and was equal to 13.55 gr.pot^− 1^, and the highest wet weight of the roots was related to the 1000 mg.kg^− 1^ treatment and was 14.36 gr.pot^− 1^, with 5.97% increase than the control, but this increase was not statistically significant. The results of the averages comparison of the effect of silica nano absorbent on roots dry weight showed that the lowest roots dry weight was related to the control treatment and was equal to 1.25 gr.pot^− 1^, which had not significant difference with the 100 and 200 mg.kg^− 1^ nano silica treatments. and the highest roots dry weight was related to 1000 mg.kg^− 1^ treatment, equal to 1.51 gr.pot^− 1^ with an increase of 20.8% compared to the control. The results showed that the effects of 500 and 1000 mg.kg^− 1^ nano silica treatments on roots dry weight were significantly different from the control treatments, 100 and 200 mg.kg^− 1^ treatments (Fig. 9).

**Fig. 10:**
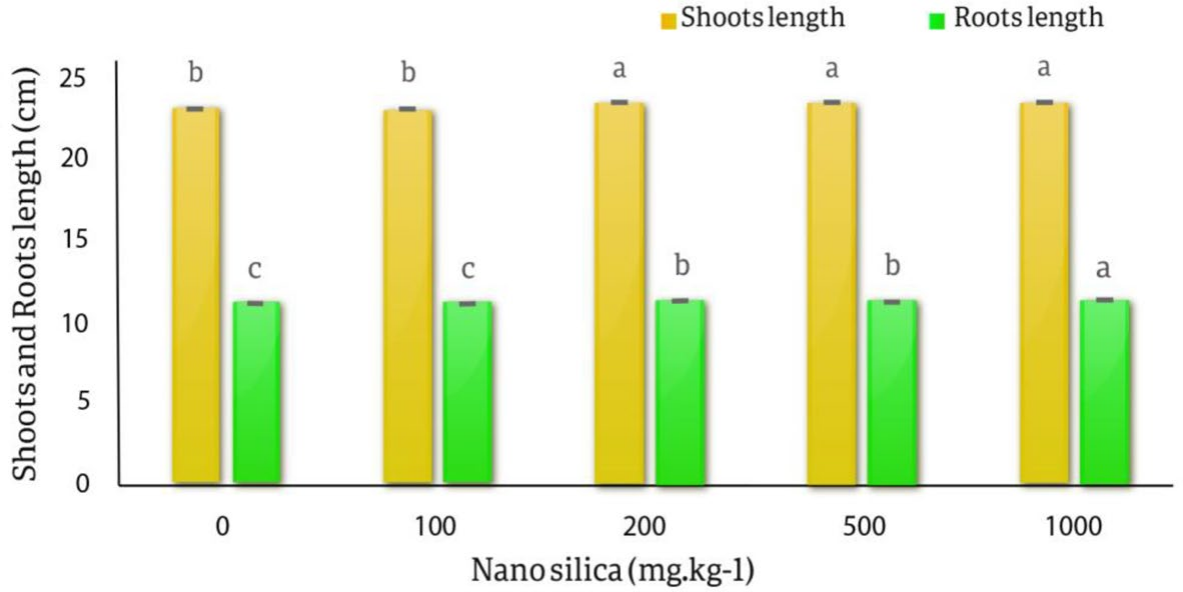
The effect of nano silica on shoots and roots length. The highest shoots were related to 200, 500 and 1000 mg.kg^− 1^ and highest rots was related to 1000 mg.kg^− 1^ treatment

The results of the averages comparison of the effect of different levels of nano silica on the length of the shoots showed that the lowest length of shoots was related to the control and 100 mg.kg^− 1^ of nano silica equal to 23.21 cm and the highest length of shoots was related to the treatment of 1000 mg.kg^− 1^ was 23.62 cm. There was a significant difference between the control treatment and 100 mg.kg^− 1^ with the 200, 500 and 1000 mg.kg^− 1^ treatments in shoots length. Also, the results of the averages comparison of the effect of various levels of nano silica on the roots length of *C. officinalis* showed that the lowest roots length was related to the control and 100 mg.kg^− 1^ treatments equal to 11.23 cm and the highest roots length was related to the 1000 mg.kg^− 1^ and was equal to 11.36 cm, there was a significant difference in different treatments for roots length (Fig. 10).

### The effect of different treatments of nano silica on phosphorus and potassium absorption by *C. Officinalis*

The results of the variance analysis of the effect of various silica nano absorbent treatments on the amount of P and K absorption in the roots and shoots showed that different levels of silica nano sorbents had a significant effect on the adsorption of P and K in the shoots and roots of *C.officinalis* in probability level of 1% (*p* < 0.01) (Table 6).

**Table 6:** Results of analysis of variance of the effects of different nano silica treatments on K and K absorption by *C. officinalis*

MS						
SOV	df	Shoots *P*	Roots *P*	Shoots K	Roots K	
Treatment	4	0.001**	0.003**	0.116**	0.074**	
Error	10	0.001	0.001	0.02	0.002	
COV (%)	-	17.74	12.8	7.99	3.46	

** and * are significant at 1% and 5% levels, respectively, and ns is not statistically significant

**Fig. 11:**
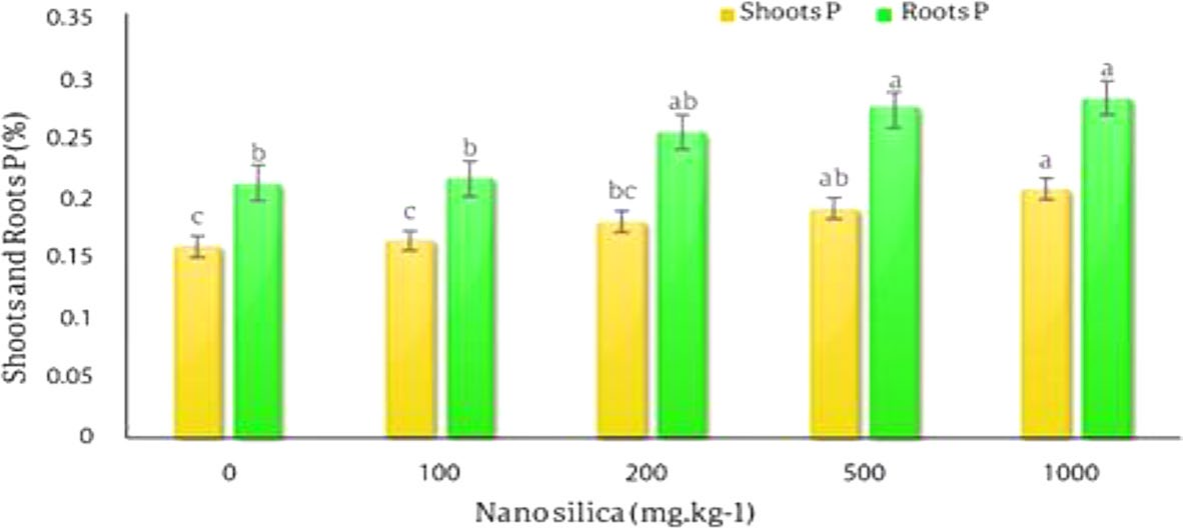
The effect of nano-silica on P amount in shoots and roots. In 1000 mg.kg^− 1^ nano silica shoots P increased 25% and roots P increased 33% compared to the control treatment

The results of the averages comparison of the effect of different levels of nano silica on the P absorption in the shoots and roots of *C. officinalis* showed with the increase in the amount of absorbent, the amount of P in the shoots and roots of the plant increased, the lowest concentration of P in the shoots was 0.16% in the control treatment and the highest amount of P was 0.2% in 1000 mg.kg^− 1^ treatment which was 25% higher than the control treatment. The lowest amount of roots P was in the control treatment equal to 0.21% and the highest amount of roots P was in 1000 mg.kg^− 1^ treatment equal to 0.284% with an increase of 33% compared to the control treatment (Fig. 11).

**Fig. 12:**
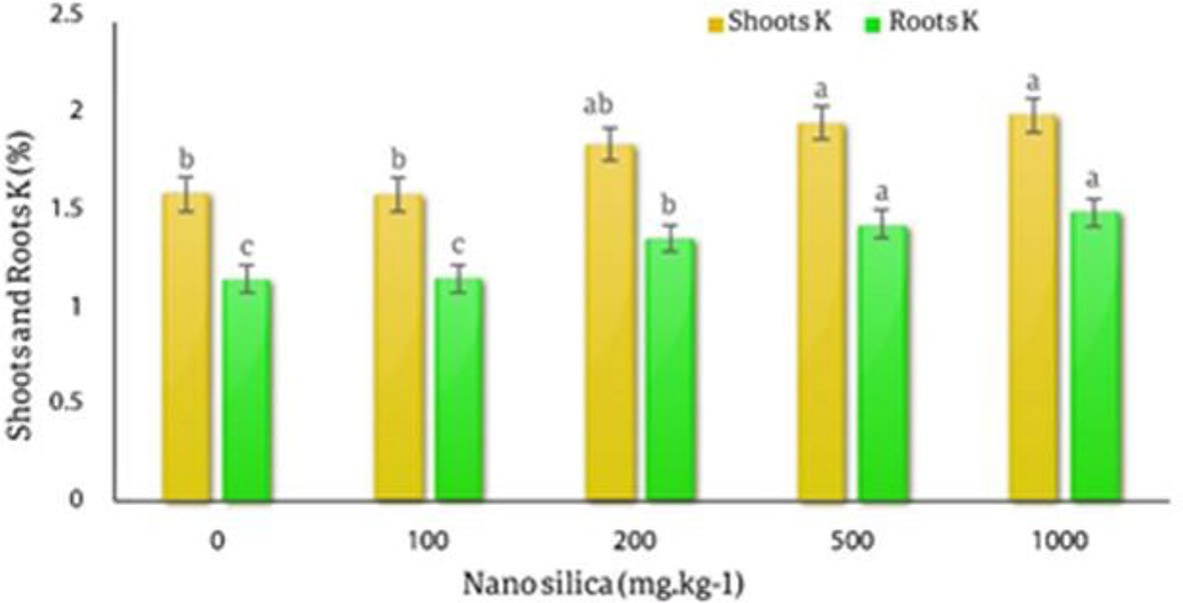
The effect of nano silica on K amount in shoots and roots. In 1000 mg.kg^− 1^ nano silica shoots K increased 26% and roots K increased 30.4% compared to the control treatment

The results of the averages comparison of the effect of different levels of nano silica on the amount of K absorption in shoots and roots of *C. officinalis* showed that with the increase in the amount of silica, the amount of K in the shoots and roots of the marigold plant increased, the lowest amount of K in the shoots was in the control treatment equal to 1.57% and the highest amount of K in the shoots was 1.98% in the treatment of 1000 mg.kg^− 1^, which was 26% higher than the control treatment. The lowest amount of roots K in the control treatment was 1.135% and the highest amount of roots K was 1.48% in the 1000 mg.kg^− 1^ treatment with an increase of 30.4% compared to the control (Fig. 12).

### The effect of nano silica on the absorption of heavy metals by the shoots and roots of the *C. Officinalis*

The results of variance analysis of the effect of different levels of silica nano absorbent on the absorption of heavy metals by the *C. officinalis* showed that different levels of silica nano absorbent had no significant effect on the amount of Zn and Ni in the roots, Pb, Cu, and Cr in the roots and shoots. There was a significant effect on Zn and Ni in shoots at the probability level of 1% (*p* < 0.01) (Table 7).

#### Lead in roots and shoots

**Fig. 13:**
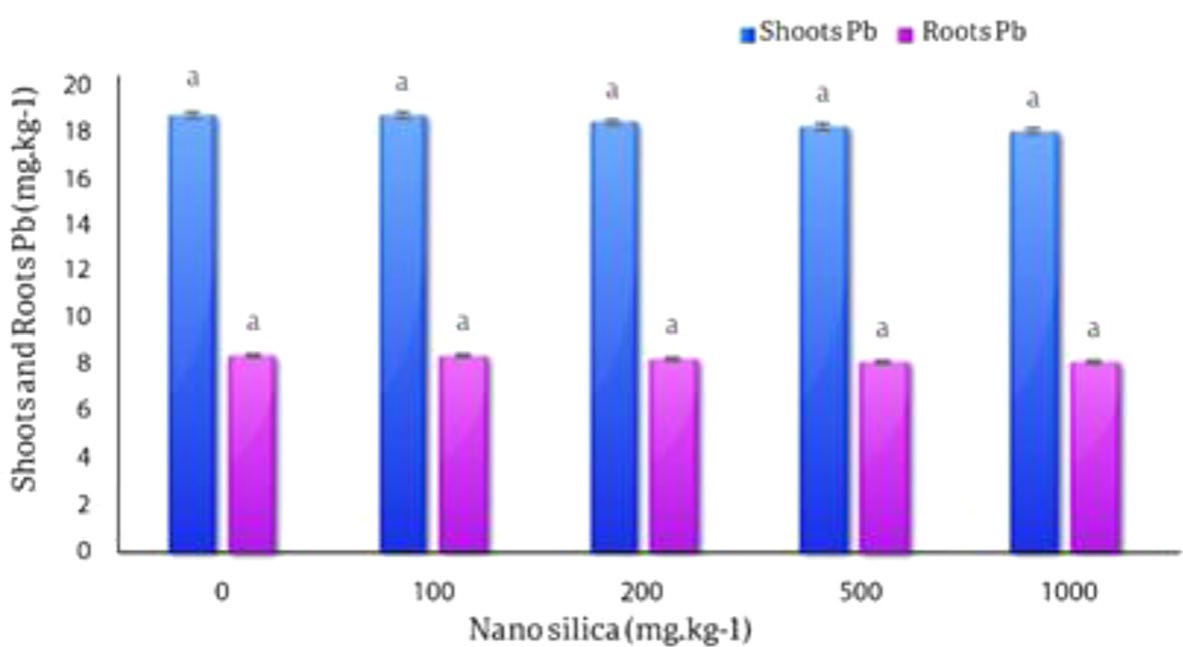
The effect of nano silica on the concentration of Pb in shoots and roots. In 1000 mg.kg^− 1^ nano silica shoots Pb decreased 4% and roots Pb decreased 3.76% compared to the control treatment

The averages comparison results of the effect of different levels of nano silica on the amount of Pb absorption by the roots and shoots of *C. officinalis* showed that there was no significant statistical difference between the treatments on the amount of Pb in the roots and shoots, although there was a difference between the treatments, so the highest amount of Pb concentration in roots observed in the control treatment (18.73 mg.kg^− 1^) and the lowest amount of Pb in roots with 3.76% decreased, was (18.05 mg.kg^− 1^) in 1000 mg.kg^− 1^ nano silica treatment. The highest concentration of Pb in shoots was observed in the control treatment (8.42 mg.kg^− 1^) and the lowest concentration of lead in the shoots with a 4% decrease was (8.1 mg.kg^− 1^) in the 1000 mg.kg^− 1^ nano silica treatment (Fig. 13).

**Table 7:** The results of variance analysis of the effects of different nano silica treatments on heavy metal absorption by *C.officinalis*

MS											
SOV	df	Root Pb	Shoot Pb	Root Zn	Shoot Zn	Root Cu	Shoot Cu	Root Cr	Shoot Cr	Root Ni	Shoot Ni
Treatment	4	0.267ns	0.068ns	19.5 ns	19.08**	1.98 ns	0.293 ns	2.7 ns	0.276ns	1.18 ns	0.289**
Error	10	0.424	0.256	5.69	1.87	0.946	0.259	1.08	0.213	0.561	0.03
COV (%)	-	3.53	6.12	4.56	2.79	5.01	6.06	4.48	4.57	4.93	2.59

** and * are significant at 1% and 5% levels, respectively, and ns is not statistically significant

#### Zinc in roots and shoots

**Fig. 14:**
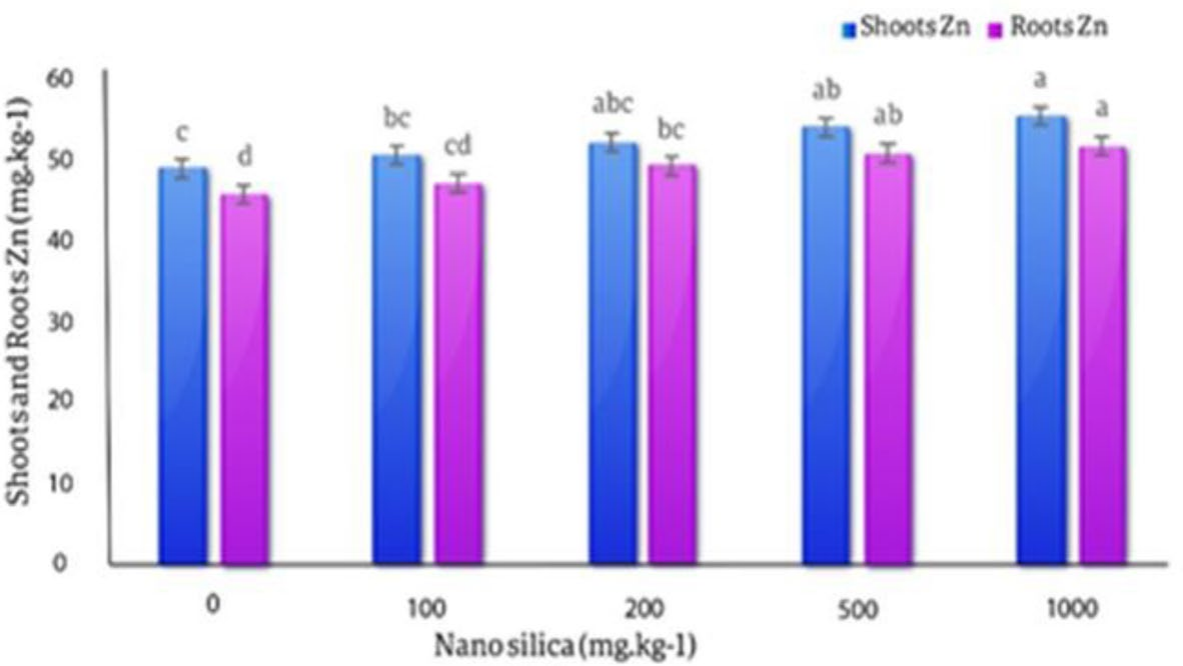
The effect of nano silica on the concentration of Zn in shoots and roots. In 1000 mg.kg^− 1^ nano silica shoots Zn 13.28% increased and roots Zn increased 13% compared to the control treatment

The averages comparison results of the effect of different levels of nano silica on the concentration of Zn in the roots showed, the lowest amount of Zn in the roots (49 mg.kg^− 1^) was in the control treatment and the highest amount of Zn in the roots (55.35 mg.kg^− 1^) was observed in the 1000 mg.kg^− 1^ treatment with 13% increase compare to control treatment, but this difference was not statistically significant. In the 1000 mg.kg^− 1^ treatment, the concentration of Zn in the roots increased by 12.96% compared to the control treatment. The results of the averages comparisons of the effect of different levels of nano silica on the amount of Zn in the shoots showed that with the increase in the amount of nano silica, the amount of Zn in the shoots increased and this difference was statistically significant. The lowest amount of Zn in the shoots of the *C. officinalis* was in the control treatment (45.77 mg.kg^− 1^) and the highest amount of Zn in shoots was (51.85 mg.kg^− 1^) in the 1000 mg.kg^− 1^ treatment with increased by 13.28% compared to the control (Fig. 14).

#### Copper in roots and shoots

**Fig. 15:**
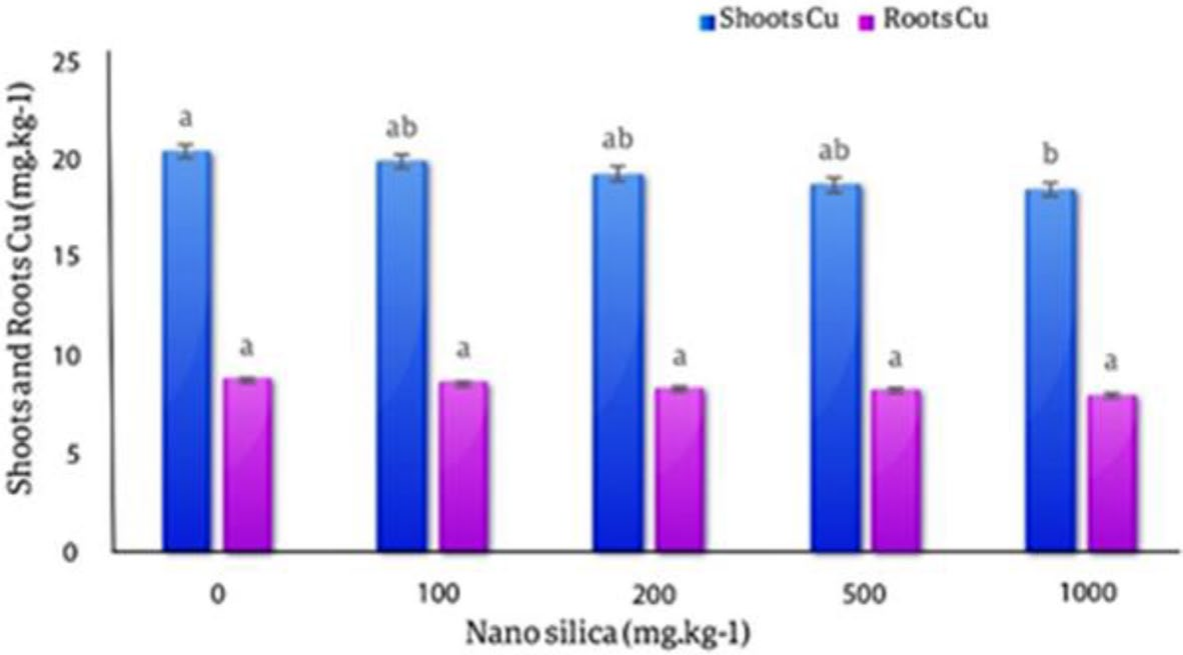
The effect of nano silica on the concentration of Cu in shoots and roots. In 1000 mg.kg^− 1^ nano silica shoots Cu decreased 9.8% and roots Cu decreased 9.44% compared to the control treatment

The results of the averages comparison of the effect of different levels of nano silica on the concentration of Cu in the roots and shoots of plant showed that with the increase in the amount of nano silica, the amount of Cu in the roots and shoots of the plant decreased. However, it was not statistically significant that the highest amount of Cu in the roots was in the control treatment (20.43 mg.kg^− 1^) and the lowest amount of Cu in the roots was observed in 1000 mg.kg^− 1^ nano silica by 9.44% decrease compared to control and equal to (18.5 mg.kg^− 1^). The highest amount of Cu in shoots (8.79 mg/kg) was observed in the control treatment and the lowest amount of Cu in shoots was observed in 1000 mg.kg^− 1^ of nano silica and equal (8 mg.kg^− 1^) with 9.8% decreased compare to control treatment (Fig. 15).

#### Chromium in roots and shoots

**Fig. 16:**
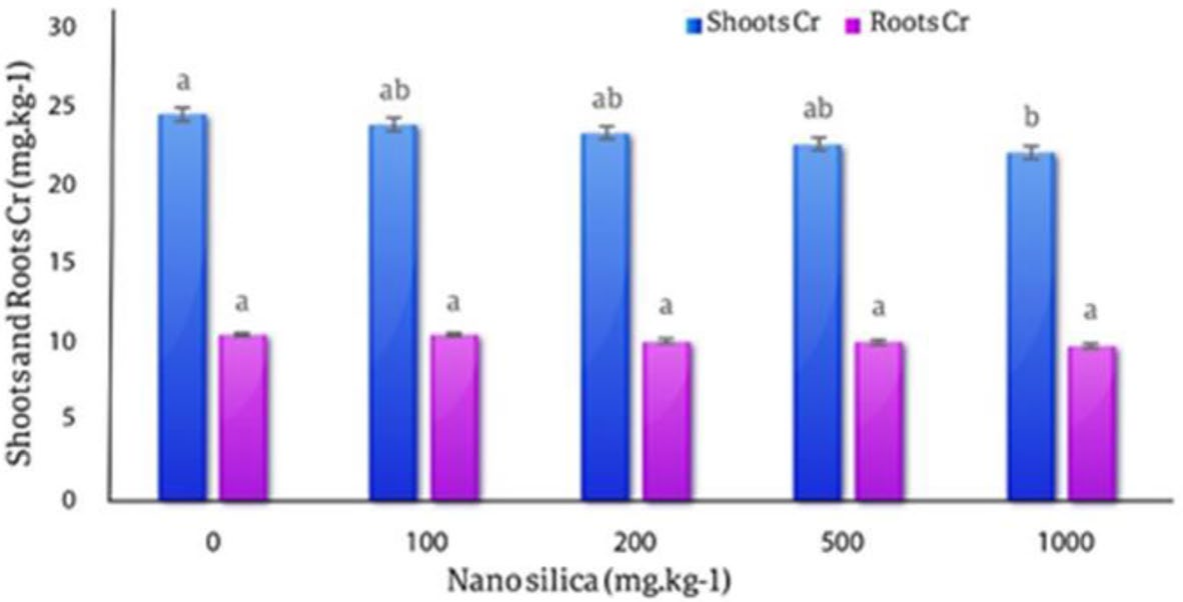
The effect of nano-silica on the concentration of Cr in shoots and roots. In 1000 mg.kg^− 1^ nano silica shoots Cr decreased 7.2% and roots Cr decreased 9.76% compared to the control treatment

The results of the averages comparison of the effect of different levels of nano silica on the amount of Cr in the roots and shoots of *C. officinalis* showed with the increase of the amount of nano silica, the concentration of Cr in the roots and shoots decreased, this difference was not statistically significant. The highest amount of Cr in the roots was in the control treatment (24.38 mg.kg^− 1^) and the lowest amount of Cr in the roots was observed in the 1000 mg.kg^− 1^ equal to (22 mg.kg^− 1^) with a decrease of 9.76% compared to the control treatment. The highest amount of Cr in the shoots (10.4 mg.kg^− 1^) was observed in the control treatment and the lowest amount of Cr in the shoots was in 1000 mg.kg^− 1^ nano silica and equal (9.7 mg.kg^− 1^) by 7.2% decreased compare to the control treatment (Fig. 16).

#### Nickel in roots and shoots

**Fig. 17:**
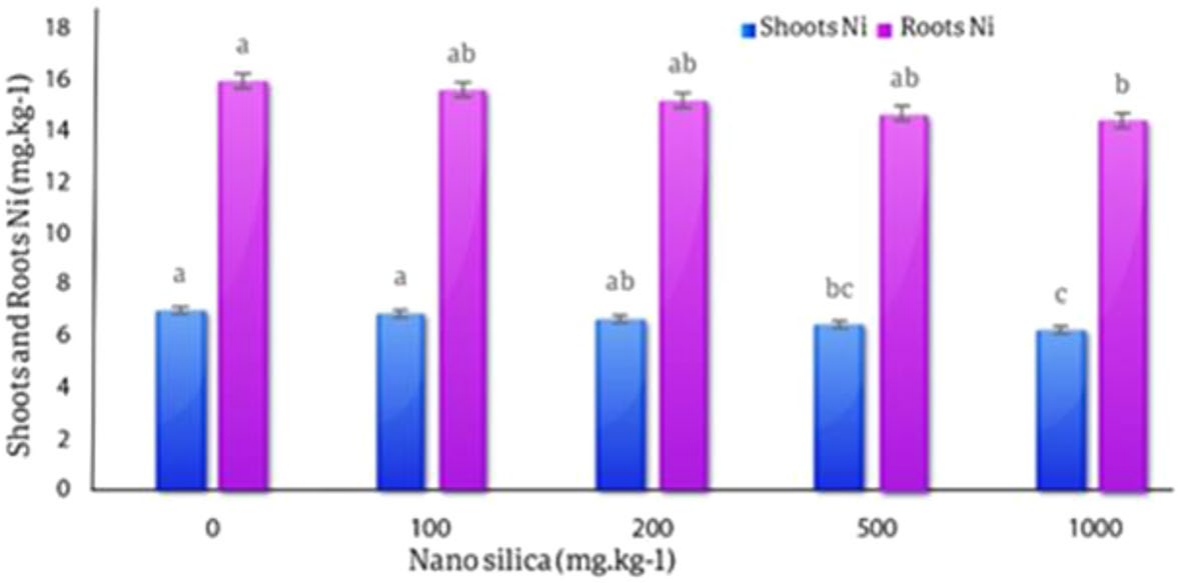
The effect of nano silica on the concentration of Ni in shoots and roots. In 1000 mg.kg^− 1^ nano silica shoots Ni decreased 9.57% and roots Ni decreased 12.5% compared to the control treatment

The results of the averages comparison of the effects of different levels of nanosilica on the amount of Ni in the roots of the plant showed that the difference between the treatments on the amount of Ni in the roots was not statistically significant, although there was a difference between the treatments, and with the increase in the amount of nano silica, the amount of Ni in the roots decreased. The highest amount of Ni concentration in roots (7.02 mg.kg^− 1^) was observed in the control treatment and the lowest amount of Ni concentration in roots (6.24 mg.kg^− 1^) was observed in the 1000 mg treatment, with a 12.5% decrease compared to control treatment. The effect of different levels of nano silica on the amount of Ni in shoots was significant, and with the increase in the amount of adsorbent, the amount of Ni in shoots decreased. The highest amount of Ni in shoots was in the control treatment equal to 15.98 mg.kg^− 1^ and the lowest amount of Ni in shoots with a decrease of 9.57% compared to the control was in the treatment of 1000 mg.kg^− 1^ and equal to 45. 14 mg.kg^− 1^ (Fig. 17).

### The effect of the different amount of nano silica on the activity of urease, dehydrogenase, and alkaline phosphatase enzymes in soil

The results of variance analysis showed that different treatments of silica nano absorbent had a significant effect at the level of 1% (*p* < 0.01) on the activity of alkaline phosphatase enzyme. Also, different silica nano absorbent treatments had a significant effect at the 5% level (*p* < 0.05) on dehydrogenase enzyme activity but did not have a significant effect on urease enzyme activity (Table 8).

**Table 8:** The results of variance analysis of the effect of the amount of silica nano absorbent on the activity of the tested soil enzymes

	MS			
		Dehydrogenase	Urease	Phosphatase
SOV	df			
Treatment	4	0.077*	7.87 ns	17.27**
Error	10	0.022	2.82	2.71
COV (%)	-	9.56	3.34	3.93

** and * are significant at 1% and 5% levels, respectively, and ns is not statistically significant

The averages comparison of the effect of silica nano absorbent on the activity of urease, dehydrogenase and alkaline phosphatase enzymes showed that the highest activity of enzymes was in the treatment of 1000 mg of nano silica/kg soil. The highest amount of urease enzyme activity was equal to 154.8 (microgram of ammonium nitrogen per gram of soil in two hours), which was 2.5% higher than the control treatment. The highest level of dehydrogenase enzyme activity with a 23.6% increase compared to the control is equal to 1.78 micrograms of triphenyl formazan per gram of soil in 16 h and the highest level of alkaline phosphatase enzyme activity is 45.45 (micrograms of p-nitrophenol per gram of soil per hour). It was 13.5% higher than the control. The greatest effect of silica nano absorbent treatment on the activity of soil enzymes was in the order of dehydrogenase > alkaline phosphatase > urease (Table 9).

**Table 9:** Comparison of the average effect of silica nano absorbent amount on the activity of soil enzymes

Nano silica (mg.kg^− 1^)	Urease	Alkaline phosphatase	Dehydrogenase
0	151.04 a	40.03 b	1.44 b
100	151.07 a	40.15 b	1.44 b
200	151.1 a	40.43 b	1.45 b
500	152.3 a	43.22 ab	1.67 ab
1000	154.8 a	45.45 a	1.78 a

‘Averages that have at least one letter in common are not statistically significant’

## Discussion

The results of this experiment showed the highest effect of nano silica in reducing the available concentration of heavy metals in the soil tested, was Pb > Cu > Zn > Ni > Cr. This variation in adsorption may be attributed to the acid-base theory, as Pb, being a hard acid, tends to complex with hydroxy groups on the silica surface more readily than Cu and Zn. This propensity for immobilization is further influenced by factors such as electric charge, hydration radius, hydration energy, and electronegativity of the ions. Pb and Cu exhibit higher adsorption due to their smaller hydrated radii (0.401 nm for Pb and 0.419 nm for Cu) and higher electronegativity compared to Zn. Unique properties exist between different heavy metal ions have such as ionic radius, electronegativity and hydration radius [45, 46]. Previous studies have shown that these intrinsic properties are inseparable from the adsorption properties of heavy metal ions, and the adsorption stability and adsorption energy are also affected by them. Although the intrinsic properties of metal ions have been used to explain the selective mechanism of heavy metal ions adsorption, no reasonable methods have been applied to prove how the adsorption selectivity is affected by these properties [47, 48]. The maximum adsorption capability of Cu^2+^ was visibly higher than that of Zn^2+^, indicating that the Cu^2+^ preferentially adsorbed to the carboxyl functional groups and occupied the active sits at the sametime. And the adsorbed Cu^2+^ was unable to be exchanged into solution by Zn^2+^. It seems that the application of 1000 mg kg^− 1^ nano silica provided sufficient surfaces for the adsorption of heavy metals thus decreasing their concentration in the soil solution. The surfaces of nano silica have hydroxyl active groups that have high adsorption capacity and are in the forms of free silanol (Si-OH) groups, free silanol diol groups (Si -(OH)_2_) and atomic bridges with oxygen ions (Si-O-Si) in surface [28]. Silanol groups on the silica surface react easily with a variety of agents. The adsorption The capacity of silica is contingent upon the charge and electronegativity of the metals present; the metal cations within the solution create chemical bonds with the siloxane oxygen groups on the surface of the silica. Both silanol and siloxane groups on the nano silica surface play crucial roles in metal adsorption capacity [49].

The results showed by increasing the soil pH, the DTPA extractable concentrations of heavy metals decreased, pH stands as a pivotal factor in regulating the equilibrium of heavy metal solutions within the soil [50]. An increase in pH precipitates a reduction in mobile metal fractions and a decline in the bioavailability of heavy metals in the soil [51]. As the solution’s pH rises, the adsorption capacity of silica for Ni, Cd, and Pb escalates [52]. The surface charge of silica amplifies with increasing pH, and at elevated pH levels, the negative charge on the silica surface enhances the absorption of metal cations [53]. As the pH of the solution increased from 4 to 6.5, the absorption of Cu by silica nanoparticles also increased. This indicates that at lower pH levels, the abundance of H^+^ ions is higher, leading to competition with other metal ions for chelation and complexation at the silica surface’s exchange sites. Conversely, at higher pH levels, the presence of hydroxyl ions increases, prompting metal ions to form hydroxides or interact with surface hydroxyl groups [54]. The extent of Cu ion absorption by silica nanoparticles depends on both the initial concentration of copper and the adsorbent itself. Silica nanoparticles (NSiO2) are particularly effective in metal ion removal due to the distinctive surface characteristics of silica [55]. Studies have demonstrated the widespread utilization of silica nanoparticles in various organic and inorganic modifications owing to their large surface area and favorable metal absorption sites. Research has also investigated the absorption of heavy metals such as Ni, Cd, and Pb by porous silica nanoparticles in aqueous environments. Nano silica exhibits a high adsorption capacity for mercury, with heterogeneous adsorption sites offering varying adsorption potentials [56].

The obtained results of this experiment showed that nano silica application leadsto increase P and K by plant and also shoots and roots dry and fresh weight in plant. The potential of silicon to improve crop yields, especially under biotic and abiotic stress conditions such as drought, salinity, and heavy metal toxicity, has been reported in various studies [57–60]. In a research conducted on the effect of silicon fertilizers on the growth, yield, and absorption of nutrients in rice plants results showed that by increasing the amount of silicon, the length of shoots, seed yield, seed weight and number Claws increased and the increase in the number of claws was evident in treatments with silica, and in the presence of silica, grain yield increased by 6% compared to the treatment without silica [61]. They also stated that in treatments with silicon, absorption of nutrients nitrogen, P, K and silicon increased in the grain and shoots of rice. Increasing the availability of P increases with the addition of silicon fertilizers [62], Also, increasing the amount of silica in the soil increases the absorption of K by the plant [63]. The application of silicon, improves K deficiency in the plant by improving the water status of the plant, through strengthening the stomatal conductivity and the rate of plant transpiration [64]. The application of silicon accelerates the growth of most plant species under normal conditions or stress, and these effects depend on the plant species and silicon concentration [65].

Results showed that applying nano silica in soil leads to decrease in heavy metal uptake by *C. officinalis*, especially in 1000 mg.kg^− 1^ nano silica treatment. Various research showed that plants that have received enough silicon, their resistance mechanism against environmental stresses and heavy metals was very high [31]. One of the important effects of silica on reducing the toxicity of heavy metals is reducing the absorption and transfer of metals in the plant. It has been reported by many researchers that the use of silica has increased the tolerance of many plant species to heavy metals by reducing their absorption and transport. The use of silica reduces the concentration of Cd in different plant species such as corn [66, 67], rice [68], wheat [69] and peanuts [70]. In research conducted on the effect of silica on the absorption of Pb metal in banana plants, results showed the treatment of 800 mg.kg^− 1^ of silica increased the pH and decreased the exchangeable Pb in the soil and the concentration of Pb in the roots and shoots were reduced. Silicon treatment caused Pb to enter the carbonate phase and the residue. 100 mg. kg^− 1^ silica treatment did not affect pH and chemical forms of Pb, but both 100 and 800 mg. kg^− 1^ silica treatments decreased the amount of Pb in banana plant vasculature. The increased tolerance of banana to Pb toxicity was related to the stabilization of Pb in the soil, and the reduction of Pb transfer from the root to shoots and the detoxification property of silica in the plant [71]. In a soil contaminated with Cr, the use of silica increased plant growth and increased the activity of POD, SOD and CAT enzymes in the plant. The use of silica changed the exchangeable forms of Cr into the forms bonded with organic matter. The application of silica increased the pH of the soil, which was one of the reasons for the reduction of Cr toxicity, and silica played the role of a modifier in Cr contaminated soils [72]. The application of silicon in soil contaminated with Cd, Zn, Pb and Cu caused immobilization of these metals in the soil and reduced their plant availability [73]. The use of silica modifiers increased the pH from 4 to 6.4 and caused a 60% decrease in the availability of metals for rice plants [74]. Shim et al. [75] reported the reduction of Pb mobility due to the use of silica. Similar results have been observed regarding Cd and Zn in contaminated soils using silicon, which has reduced the bioavailability of metals by forming more stable fractions [76]. Gupta et al. [77] reported that the use of silicon in copper-stressed wheat caused the formation of Cu complexes with organic acids and prevented the transfer of Cu to aerial organs. The use of diatomite as a silicon source reduced the toxicity of Cd in wheat and reduced the available Cd in the soil [78]. The external application of silica reduced visible stress symptoms including (low biomass and leaf chlorosis) under Cu stress [68]. Many studies have been conducted on the relationship between silica and plant tolerance to heavy metals [5, 7, 9, 41]. The main mechanisms of stress correction of heavy metals by silicon in plants include: **(1)** entanglement or combination of metals with silicon, **(2)** preventing the transfer of metals from roots to buds and aerial organs, **(3)** division Fixing metal ions inside the plant, (4) stimulating the antioxidant system and changing the cellular structure in plants [79]. One of the important roles of silica in metal detoxification is related to changing plant cell mechanisms and biochemical interactions with the external growth environment [80]. The positive effects of silicon are different in various plant species and are usually seen more in plants that absorb a higher concentration of silicon [81]. The silicon mechanisms for modulating the stresses of heavy metals are divided into internal and external mechanisms [27]. In external mechanisms, silica modulates the toxicity of heavy metals through various methods such as reducing the absorption or activity of the metal, or changing the chemical form of the metal, or increasing the pH. In internal mechanisms, silica reduces the negative effects of heavy metals in various ways such as stimulating the activity of antioxidant enzymes, complexing with heavy metals and cell wall changes and controlling metal transfer [11]). Williams and Vlamis [73] observed for the first time that the effect of silicon on the reduction of manganese (Mn) toxicity in the atmosphere was not a result of reducing the concentration of Mn in the soil, but it was due to its confinement in the plant tissue. Another type of metal sequestration by silicon in the plant is related to the transport activities that lead to an increase in the concentration in the roots compared to the aerial organs [82]. The application of silica in soil contaminated with Cd reduced the transfer of Cd from roots to seeds and aerial organs of wheat [69]. Barcelo et al. [83] observed a significant increase in malic acid under the application of silica in plants grown in aluminum (Al)contaminated soil. They stated that the reduction of Al toxicity was related to the chelation of Al with malic acid. The transfer of Cu from the roots to the aerial organs of wheat occurred under the use of silicon, which could be due to the increase in the ratio of citrate, malate in wheat roots [82]. These studies showed that silicon may indirectly improve the chelation of heavy metals in plants and reduce their toxicity. Also, silicon has a protective role against various stresses and causes the accumulation of polysalicylic acid inside the plant, which increases the resistance of the plant [84]. Many researchers reported that co-precipitation of silica with heavy metals reduced the concentration of toxic ions in plants [85]. Another mechanism of silica in modulating the toxic effects of heavy metals for plants is the change of plant structure under heavy metal stress conditions. Silica and its availability improve the morphological characteristics and anatomical dimensions of the plant and help to overcome the side effects of heavy metal stress. An increase in height, root length, number, and size of leaves has been observed due to the application of silicon in plants under Pb, Zn and Cd stress [60]. Silica and Cr treatment increased plant Pb and number of claws, root length and leaf size of barley compared to the control [86]. Silicon increased the thickness of the epidermal layer of maize leaves under Mn stress [87]. Also, the use of silicon has increased the thickness of wood vessels under the stress of Cd and Zn [88]. Co-precipitation of silica with metals also leads to reduction of heavy metal stress in plants. Zhang et al. [89] stated that under Cr stress, silica reduces Cr absorption by plants. They stated that silica increases root secretions that can chelate metals and reduce their availability. Silicon causes the deposition of lignin in the cell wall, which causes metal ions to bond with the cell wall and reduces the transfer of metal to other parts [90]. The application of silicon increased the concentration of Zn, iron (F) and Mn in the roots of corn, wheat and carrot plants, but decreased the concentration of Cu and Zn in the aerial parts of these plants [91]. Silica increased the tolerance of cucumber plants to Mn toxicity, which was caused by the strong binding of Mn to the cell wall and the decrease in the amount of symplastic Mn due to the use of silica [92]. Silicon has significantly reduced the absorption and displacement of Pb in different varieties of rice [11]. The role of silica as a physical inhibitor in plants can also be considered as a hypothesis in the reduction of Pb absorption by plants in the presence of silica [27].

high concentration of heavy metals in the soil damages the growth of crops [93]. The results showed nano silica had positive effect on soil enzyme activity, and application of nano silica leads to increase the enzymes activity in treated soils. The number, diversity and activity of soil microbes and thus the production of extracellular enzymes is reduced by heavy metals [10]. High Cd concentrations significantly reduced the activity of catalase, urease, dehydrogenase, cellulase and phosphatase enzymes [11]. researchers have shown that soil enzymes are more sensitive to stress of heavy metals than plants and animals [82, 94]. The conducted studies indicate that silicon dioxide nanoparticles can be used as an adsorbent to remove natural pollutants and metal ions [30]. The reason for achieving high removal efficiency of metal ions at the level of silicon nanoparticles is as follows: Silica surface characteristics, metal ion adsorption kinetics, changes in pH, temperature and pressure on metal ions, desorption of metal ions from a solid mixture and the efficiency of bonds on the surface of silicon [95, 96]. The results of various researches have shown that silica has been very successful in absorbing metals from the environment, this high efficiency in absorbing metals is related to characteristics such as high surface area, having active absorption centers, which increases the selectivity and the transfer rate is high inside the cavity structure [97].

## Conclusion

In conclusion, the study on the “Role of Nano Silica as a Soil Modifier in Alleviating Heavy Metal Stress for *C. officinalis*” provides valuable insights into the promising application of nano silica in mitigating heavy metal stress in the growth of *C. officinalis.* The findings underscore the efficacy of nano silica in improving soil conditions and promoting the overall health and vitality of the plant under heavy metal stress. The results demonstrate that nano silica serves as an effective soil modifier, enhancing nutrient availability and reducing the toxic effects of heavy metals on *C. officinalis*. This results, suggesting that nano silica can play a pivotal role in minimizing the adverse impacts of heavy metal contamination on plant growth. Furthermore, the study sheds light on the mechanisms underlying the positive effects of nano silica, emphasizing its ability to modulate key physiological and biochemical processes in *C. officinalis*. But according to the results of this research, using nano silica leads to increase soil pH, so it is necessary to investigate use of nano silica in various type of soils, To prevent possible harmful effects in different soils. Therefore, it seems necessary to investigate nano silica in different types of soil in terms of texture, structure, salinity, acidity and also the amount of organic matter. Although nano silica is effective in cleaning the environment from pollutants, one should also be aware of its effects on the physical, chemical and biological conditions of the soil.

While the findings are promising, it is essential to acknowledge the need for further research and field trials to validate the long-term effectiveness and environmental safety of nano silica as a soil modifier. Additionally, exploring its application in diverse plant species and under varying environmental conditions will contribute to a comprehensive understanding of its broader implications. Nano silica, particularly at a concentration of 1000 mg.kg^− 1^, enhanced roots and shoots length and dry weight. Moreover, it increased phosphorus and potassium concentrations in plant tissues while decreasing heavy metals uptake by plant. Also using nano silica leads to increasing soil enzymes activity in soil.

## Data Availability

The datasets used and/or analysed during the current study are available from the corresponding author Maryam Samani. All data generated or analysed during this study are included in this manuscript.
